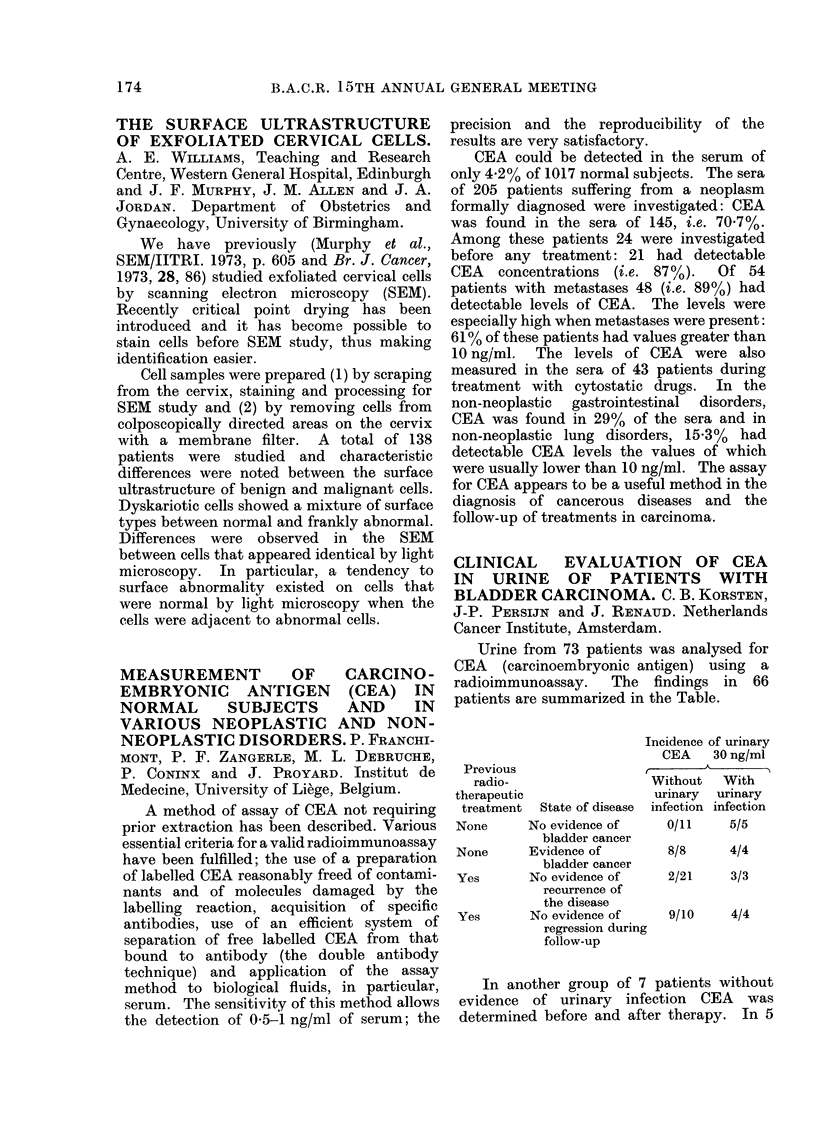# Proceedings: Measurement of carcinoembryonic antigen (CEA) in normal subjects and in various neoplastic and non-neoplastic disorders.

**DOI:** 10.1038/bjc.1974.136

**Published:** 1974-08

**Authors:** P. Franchimont, P. F. Zangerle, M. L. Debruche, P. Coninx, J. Proyard


**MEASUREMENT OF CARCINO-EMBRYONIC ANTIGEN (CEA) IN NORMAL SUBJECTS AND IN VARIOUS NEOPLASTIC AND NON-NEOPLASTIC DISORDERS.** P. FRANCHIMONT, P. F. ZANGERLE, M. L. DEBRUCHE, P. CONINX and J. PROYARD. Institut de Medecine, University of Liège, Belgium.

A method of assay of CEA not requiring prior extraction has been described. Various essential criteria for a valid radioimmunoassay have been fulfilled; the use of a preparation of labelled CEA reasonably freed of contaminants and of molecules damaged by the labelling reaction, acquisition of specific antibodies, use of an efficient system of separation of free labelled CEA from that bound to antibody (the double antibody technique) and application of the assay method to biological fluids, in particular, serum. The sensitivity of this method allows the detection of 0·5–1 ng/ml of serum; the precision and the reproducibility of the results are very satisfactory.

CEA could be detected in the serum of only 4·2% of 1017 normal subjects. The sera of 205 patients suffering from a neoplasm formally diagnosed were investigated: CEA was found in the sera of 145, *i.e.* 70·7%. Among these patients 24 were investigated before any treatment: 21 had detectable CEA concentrations (*i.e.* 87%). Of 54 patients with metastases 48 (*i.e.* 89%) had detectable levels of CEA. The levels were especially high when metastases were present: 61% of these patients had values greater than 10 ng/ml. The levels of CEA were also measured in the sera of 43 patients during treatment with cytostatic drugs. In the non-neoplastic gastrointestinal disorders, CEA was found in 29% of the sera and in non-neoplastic lung disorders, 15·3% had detectable CEA levels the values of which were usually lower than 10 ng/ml. The assay for CEA appears to be a useful method in the diagnosis of cancerous diseases and the follow-up of treatments in carcinoma.